# Results of an Interlaboratory Comparison of a Liquid Chromatography–Isotope Ratio Mass Spectrometry Method for the Determination of ^13^C/^12^C Ratios of Saccharides in Honey

**DOI:** 10.1093/jaoacint/qsab091

**Published:** 2021-09-22

**Authors:** Eric Aries, Oliver De Rudder, Georgios Kaklamanos, Alain Maquet, Franz Ulberth

**Affiliations:** European Commission, Joint Research Centre, Retieseweg 111, 2440 Geel, Belgium

## Abstract

**Background:**

Stable carbon isotope analysis of sugars in honey by LC–isotope ratio mass spectrometry (IRMS) is a useful tool for detecting adulteration of honey with extraneous sugar. Purity criteria based on ^13^C/^12^C ratios of saccharides in honey, determined by LC–IRMS of a large number of authentic honey samples, have been elaborated. However, no interlaboratory comparison (ILC) has yet been performed to estimate the precision of the method under reproducibility conditions.

**Objective:**

To address this knowledge gap an ILC involving 14 laboratories and using six honey samples was conducted.

**Methods:**

The participants were allowed to use their LC–IRMS-based method of choice for sample preparation and compound separation.

**Results:**

The precision figures were estimated according to ISO 5725:1994. The repeatability relative standard deviation (RSD_r_) for the determination of δ^1^^3^C values of fructose and glucose varied between 0.3 and 0.5%, with 0.3 and 1.0% for disaccharides, and 0.7 and 2.8% for trisaccharides. The RSD_R_ varied between 0.8 and 1.8% for the monosaccharides, 1.0 and 1.5% for disaccharides, and 1.4 and 2.8% for trisaccharides.

**Conclusion:**

Based on the obtained precision data the LC–IRMS method for the determination of ^13^C/^12^C ratios of saccharides in honey was considered fit for the conformity assessment of honey with established purity criteria.

**Highlights:**

Precision estimates for a LC–IRMS method to determine ^13^C/^12^C ratios of saccharides in honey were obtained through an ILC. The data created can form the basis for the standardization of the method by interested standards-developing organizations for use in official control.

Honey is a natural sweetener with attractive sensory properties. Certain honey types can offer health benefits as well. Demand has increased over the years, partially due to population increase but also due to consumers’ preference for natural and unprocessed food (1). It is a globally traded commodity following complex trade routes, which makes quality and authenticity control difficult. Honey is among the commodities most vulnerable to fraud (2); it was listed among the top 10 products that are most at risk of food fraud in the “Report on the Food Crisis, Fraud in the Food Chain and the Control Thereof” of the European Union (EU) Parliament (3). A marketing standard for honey exists in the EU, laying down composition criteria for honey (4). A set of analytical methods have been standardized by the International Honey Commission (5), which allows enforcement of the provisions of the Directive. Those methods are widely used but are not always appropriate for assessing the authenticity of the product. More sophisticated methods using chromatographic and/or spectroscopic techniques have been developed to verify claims made on the label in relation to geographical and/or floral origin. However, adulteration of honey by nondeclared dilution with foreign sugars/sweeteners is likely among the most frequently encountered cases. The current state of development of detection methods has been reviewed earlier (6), and more recently by other authors (7–9).

Syrups that mimic the composition of honey that are produced by chemical and/or enzymatic modification of starch or sucrose are difficult to detect (10). If the starting product is obtained from a C4 plant, such as maize or sugar cane, stable carbon isotope ratio analysis (SCIRA) using a combination of an elemental analyzer (EA) and an isotope ratio mass spectrometer (IRMS) offers a possibility to detect additions down to a level of 7% (11). Sugars originating from C3 plants such as beet root or generated from rice or wheat starch escape detection by SCIRA. Combining LC with IRMS (LC–IRMS) offers new possibilities for detecting honey adulteration with sugars derived from C3 plants and increases the sensitivity for detecting C4 sugars (12, 13). Addition of 1% C4 sugars and 10% C3 sugars can be reliably detected using the LC–IRMS approach. The method has gained popularity (14–20) but has never been subjected to multilaboratory validation, which is a prerequisite for further developing it into a standard by a standards-developing organization.

The objective of this work was to carry out an interlaboratory comparison (ILC) to obtain precision data for the LC–IRMS determination of ^13^C/^12^C ratios of sugars in honey, which can be used to detect adulterated honey. The study participants were not required to follow a fixed standard operating procedure (SOP) because settings for operating the LC–IRMS instrumentation would inevitably vary depending on the equipment. In addition, as organic solvents/compounds cannot be used in LC–IRMS, a limited number of possibilities exist for the chromatographic separation of sugars in honey. With a few exceptions, only polymeric styrene–divinylbenzene resins loaded with cations (H^+^, Ag^+^, Ca^2+^, Pb^2+^) as the stationary phase and high-purity water as the mobile phase are employed. Sample preparation is also straightforward and consists just of diluting honey with water, followed by filtration.

## Experimental

### Materials

Samples for the ILC were selected from the Joint Research Centre of the European Commission collection of honeys; all of them were analyzed by EA/LC–IRMS (13) and for the presence of oligosaccharides using an in-house-developed ultra-high-performance LC (UHPLC–MS) method (Table 1). Bulk honey samples were warmed in a water bath to 40°C, homogenized by stirring, and aliquots of 2 g were filled into screw-cap glass vials, coded, and stored at room temperature in the dark until dispatch. Homogeneity was not studied as honey is a liquid, although viscous, but stirring at an elevated temperature should ensure proper homogeneity. Samples were dispatched by overnight courier.

**Table 1.** Characteristics of honey samples used in the interlaboratory comparison

Sample	Characteristic	Indicated by
A (monofloral—lemon)	Adulterated	Presence of C4 sugars by EA/LC–IRMS
B (polyfloral)	Genuine	Conformity with purity criteria (13) and absence of oligosaccharides
C (honeydew)	Undecided	Presence of oligosaccharides by LC–IRMS but otherwise conforming with purity criteria (13)
D (honeydew)	Adulterated	Presence of oligosaccharides by LC–IRMS and UHPLC–MS
E (monofloral—acacia)	Undecided	Different δ ^13^C observed in the trisaccharide fraction
F (Monofloral—lavender)	Genuine	Conformity with purity criteria (13) and absence of oligosaccharides

**Table 2.** Experimental conditions used by the interlaboratory comparison participants

Lab	Column	Flow rate, mL/min	Method according to
1^a^	Shodex SUGAR Series 7 µm, 20 Å, 300 × 8 mm (pre-column: Shodex Guard Column, 8 µm SP-G Spherical Polymer, 50 × 6 mm)	0.50	(13)
2	Agilent Hi-Plex Ca column, 7.7 × 300 mm (pre-column: Agilent Hi-Plex Ca column, 7.7 × 50 mm)	0.60	(13)
3	Phenomenex Rezex RCM-Monosaccharide Ca^2+^ (8%), 300 × 7.8 mm	0.45	(13)
4	Phenomenex Rezex Ca^2+^, 300 × 8 mm	0.30	(13)
5	Phenomenex Rezex RCM-Monosaccharide Ca^2+^ (8%)	0.40	(13)
6^b^	Repromer H+, Dr. Maisch (pre-column: Repromer H+, Dr. Maisch)	0.25	
7	ThermoFisher HyperREZ XP Carbohydrate H+, 8 μm, 300 × 8 mm	0.40	(12)
8	Alltech 700CH carbohydrate column	0.40	(20)
9	Ca^2+^ column, 8 µm, 300 × 7.8 mm (pre-column: Ca^2+^ column, 8 µm, 10 × 7.8 mm)	0.30	(13)
10	ThermoFisher CarboPac PA1	0.20	
11	Phenomenex Rezex RCM-Monosaccharide Ca^2+^, 300 × 7.8 mm	0.30	(13)
12	Waters Sugar-Pak I , 10 μm, 300 × 6.5 mm	0.50	(13)
13	No details reported		
14	Phenomenex Rezex RCM-Monosaccharide Ca^2+^, 300 × 7.8 mm	0.30	(14)

a Collection of sugar fractions, followed by EA-IRMS.

b Thermal conversion interface.

Each ILC participant received one vial per honey sample together with instructions for performing the analyses and a reporting template. Each sample had to be analyzed in duplicate and the d^13^C values (in ‰) for fructose, glucose, disaccharides, trisaccharides, and oligosaccharides had to be reported, together with details of the chromatographic separation step.

### Methods

The LC–IRMS method for the compound-specific determination of ^13^C/^12^C ratios is based on the separation of the sugars in honey by a form of ion interaction chromatography, mostly combined with a size-exclusion mechanism if porous resins are used. The column effluent is fed into an interface where organic compounds are oxidized to CO_2_. CO_2_ isotopes with *m/z* 44, 45 and 46 are separated in the spectrometer and detected using Faraday cups, and compound-specific δ^13^ C (‰) values are calculated as [(^13^C/^12^C sample − ^13^C/^12^C VPDB) × 1000] ÷ ^13^C/^12^C VPDB, where VPDB = Vienna Pee Dee Belemnite, the reference substance.

Fourteen laboratories participated in the ILC. Details of the experimental conditions used are summarized in Table 1. Most laboratories employed polymeric styrene–divinylbenzene resins in the Ca^2+^ form and followed the protocol as described in (13); one participant used an anion-exchange (CarboPac) column. With one exception, all laboratories used an interface where the separated sugars were converted by wet oxidation with sodium peroxodisulfate and ortho-phosphoric acid to CO_2_; one participant used a thermal conversion interface. One participant used an off-line method by collecting the sugar fractions separated by LC, vacuum-dried them, and analyzed them by EA-IRMS.

Statistical evaluation of the ILC data followed the ISO 5725:1994 procedures (21); computations were done with the PROLab Plus software package (QuoData, Dresden, Germany).

## Results

The data reported back by the participants were checked for consistency with the ILC instructions, plotted and visually examined for irregularities. Next, for each data set (d^13^C values for sugar *x* in honey sample *y*) outliers were identified by applying Cochran and Grubbs/Double Grubbs tests. After removing the outlying data, the mean, repeatability SD (s_r_), reproducibility SD (s_R_), their relative values, and repeatability (r) and reproducibility (R) were computed for each data set. The results are summarized in Tables 3–6. With two exceptions, the repeatability relative standard deviation (RSD_r_) was less than 1% and the RSD_R_, was less than 2% for all samples and sugar types although the participants did not follow a prescribed procedure and used different spectrometer brands (Elementar, Nu Instruments, ThermoFisher). In addition, the results of the participant using a thermal conversion instead of a wet oxidation interface and the one using a CarboPac instead of a polymeric styrene–divinylbenzene column for sugar separation were not identified as outliers, with one exception: one result reported by the participant using the thermal conversion interface was identified as a Double Grubbs outlier.

**Table 3.** Precision data for δ^13^C of fructose determined by LC–IRMS in honey samples

Parameter	Sample					
	A	B	C	D	E	F
Number of laboratories	14	14	14	14	14	14
Number of laboratories after elimination of outliers	14	13	13	14	14	14
Outliers (Cochran test)	—^a^	1	1	—	—	—
Mean (δ^13^C‰)	−24.33	−25.07	−24.40	−24.26	−24.49	−26.41
Reproducibility standard deviation (s_R_)	0.38	0.28	0.19	0.27	0.38	0.37
Repeatability standard deviation (s_r_)	0.13	0.09	0.09	0.08	0.08	0.12
Relative s_R_ (%)	1.6	1.1	0.8	1.1	1.5	1.4
Relative s_r_ (%)	0.5	0.3	0.4	0.3	0.3	0.5
Reproducibility, R (2.80 * s_R_)	1.08	0.79	0.53	0.75	1.05	1.04
Repeatability, r (2.80 * sr)	0.36	0.24	0.24	0.23	0.21	0.33

a No outlier was observed.

**Table 4.** Precision data for δ^13^C of glucose determined by LC–IRMS in honey samples

Parameter	Sample					
	A	B	C	D	E	F
Number of laboratories	14	14	14	14	14	14
Number of laboratories after elimination of outliers	13	14	12	13	14	14
Outliers (Cochran test)	—^a^	—	1	—	—	—
Outliers (Grubbs test)	1	—	1	1	—	—
Mean (δ^13^C‰)	−24.43	−24.47	−23.58	−23.71	−24.53	−26.03
Reproducibility standard deviation (s_R_)	0.31	0.43	0.34	0.33	0.29	0.42
Repeatability standard deviation (s_r_)	0.10	0.10	0.12	0.13	0.12	0.12
Relative s_R_ (%)	1.3	1.8	1.5	1.4	1.2	1.6
Relative s_r_ (%)	0.4	0.4	0.5	0.5	0.5	0.5
Reproducibility, R (2.80 * s_R_)	0.85	1.20	0.96	0.92	0.81	1.18
Repeatability, r (2.80 * sr)	0.27	0.29	0.33	0.36	0.32	0.33

a No outlier was observed.

**Table 5.** Precision data for δ ^13^C of disaccharides determined by LC–IRMS in honey samples

Parameter	Sample					
	A	B	C	D	E	F
Number of laboratories	13	13	13	13	13	13
Number of laboratories after elimination of outliers	13	13	13	11	13	13
Outliers (Double Grubbs test)	—^a^	—	—	2	—	—
Mean (δ^13^C ‰)	−24.85	−26.27	−25.23	−25.13	−24.88	−27.01
Reproducibility standard deviation (s_R_)	0.35	0.34	0.36	0.24	0.36	0.31
Repeatability standard deviation (s_r_)	0.18	0.08	0.24	0.15	0.14	0.12
Relative s_R_ (%)	1.4	1.3	1.4	1.0	1.45	1.1
Relative s_r_ (%)	0.7	0.3	1.0	0.6	0.6	0.4
Reproducibility, R (2.80 * s_R_)	0.99	0.96	1.01	0.67	1.01	0.86
Repeatability, r (2.80 * sr)	0.49	0.23	0.68	0.41	0.39	0.34

a No outlier was observed.

**Table 6.** Precision data for δ^13^C of trisaccharides determined by LC–IRMS in honey samples

Parameter	Sample					
	A	B	C	D	E	F
Number of laboratories	10	13	13	13	10	10
Number of laboratories after elimination of outliers	10	12	13	13	9	10
Outliers (Grubbs test)	—^a^	1	—	—	—	—
Outliers (Cochran test)	—	—	—	—	1	—
Mean (δ^13^C ‰)	−24.05	−26.74	−24.69	−25.18	−22.73	−25.73
Reproducibility standard deviation (s_R_)	0.68	0.36	0.35	0.50	0.45	0.57
Repeatability standard deviation (s_r_)	0.68	0.19	0.23	0.25	0.15	0.38
Relative s_R_ (%)	2.8	1.4	1.4	2.0	2.0	2.2
Relative s_r_ (%)	2.8	0.7	0.9	1.0	0.7	1.5
Reproducibility, R (2.80 * s_R_)	1.90	1.02	0.98	1.40	1.26	1.59
Repeatability, r (2.80 * sr)	1.90	0.52	0.64	0.70	0.42	1.07

a No outlier was observed.

For sample A two participants reported larger differences for their duplicate analyses of trisaccharides, which, due to masking effects, were not flagged by the Cochran test as outliers and were kept for further data evaluation. Consequently, the estimate for the between-laboratory component of reproducibility (s_L_) could not be calculated by analysis of variance and, therefore, s_R_ was set equal to s_r_ for this sample. This explains the rather high precision estimates for this sample (Table 6).

## Discussion

Isotope ratios of light elements are widely used to verify the authenticity of various food commodities, typically for verifying geographical origin or detecting the extension/dilution of food originating from plants using the Calvin–Benson photosynthetic cycle (C4 plants) by those using the Hatch–Slack pathway (C3 plants). SCIRA by EA-IRMS has been in use for many years for this purpose, and single- and multilaboratory validation data are available. However, this is not the case for LC–IRMS, where only repeatability data obtained in single laboratories have been published.

Usually, the main goal of a validation ILC is to conclude whether the method is fit-for-purpose based on the obtained precision data, which are judged against a benchmark. The Horwitz ratio, the relation of the RSD_R_ obtained in the ILC to the corresponding predicted value derived from an empirical equation, is often used for this purpose (22). However, this approach cannot be applied, as the Horwitz equation has been estimated for measurands expressed as mass ratios, whereas for IRMS-based methods the measurand is expressed as an isotope ratio. Consequently, literature data were used to assess the fitness-for-purpose of the assessed LC–IRMS method.

Precision data for AOAC Official Method **998.12** (on C4 plant sugars in honey by EA-IRMS) (11) were obtained by an interlaboratory study, with a reported repeatability standard deviation (s_r_) of 0.06‰ and reproducibility standard deviation (s_R_) of 0.14‰. The Forensic Isotope Ratio Mass Spectrometry (IRMS) network organized a number of ILCs using different test items and concluded that s_r_ is satisfactory (median δ^13^C of 0.07‰ for six ILCs), but s_R_ (median δ^13^C of 0.20‰ for six ILCs) should be improved through the use of certified reference materials for calibration and method standardization (23). The average s_r_ and s_R_ values were 0.1 and 0.2‰ for δ^13^C values, respectively, for hard cheeses obtained in an ILC (24), which is in good agreement with the previously reported data.

Precision data for compound-specific SCIRA, in particular for LC–IRMS, are less abundant. The seminal work of Cabañero et al. (12) lists a single-laboratory repeatability standard deviation of 0.2‰ for δ^13^C fructose, glucose and disaccharides, and Elflein and Raezke (13) reported 0.1‰ for fructose and glucose, 0.3‰ for disaccharides, and 0.5‰ for trisaccharides.

In the present study the repeatability standard deviation of δ^13^C values obtained by a multilaboratory ILC was in the same range as found by single-laboratory validation. Values between 0.09 and 0.13‰ for monosaccharides, 0.08 and 0.24‰ for disaccharides, and 0.36 and 0.68‰ for trisaccharides were obtained.

As expected, values for reproducibility standard deviations were higher by a factor of 3–4 (0.19–0.43‰ for monosaccharides, 0.24–0.36‰ for disaccharides, and 0.36–0.68‰ for trisaccharides), which reflects the differences between the participating laboratories regarding testing conditions and equipment. No SOP was prescribed and the participants were free to use their sample preparation and separation method of choice. It was possible to grant this freedom as LC–IRMS excludes the use of carbon-containing mobile phases and this restricts the available options for separating saccharides to macroporous resins and high-purity water as eluent. The reproducibility standard deviation values are higher compared with published s_R_ values obtained by EA-IRMS; however, as compound-specific SCIRA requires a separation step, which will inevitably introduce additional variation, the precision data obtained in the present ILC are deemed adequate and the LC–IRMS method for the compound-specific SCIRA of mono-, di-, and trisaccharides in honey is fit-for-purpose. The method reproducibility standard deviation can be used to estimate measurement uncertainty according to ISO 21748:2017 (25), which, in turn, should be used when assessing honey for compliance with the purity criteria established by Elflein and Raezke (5).
